# The *Shank3-InsG3680(*+/+) mouse model of autism spectrum disorder displays auditory avoidance in a novel behavioral test

**DOI:** 10.3389/fnbeh.2023.1205507

**Published:** 2023-08-24

**Authors:** Ana Margarida Gonçalves, Nuno Sousa, Luis Jacinto, Patricia Monteiro

**Affiliations:** ^1^Life and Health Sciences Research Institute, School of Medicine, University of Minho, Braga, Portugal; ^2^ICVS/3B’s—PT Government Associate Laboratory, Braga/Guimarães, Portugal; ^3^Experimental Biology Unit, Department of Biomedicine, Faculty of Medicine, University of Porto (FMUP), Porto, Portugal

**Keywords:** autism spectrum disorder (ASD), *Shank3*, auditory hypersensitivity, behavior, sensory alterations, animal model

## Abstract

**Introduction:**

Autism spectrum disorder (ASD) is characterized by deficits in communication and social interaction, restricted interests, repetitive behaviors, and sensory alterations, with auditory hypersensitivity being one of the most commonly reported sensory–perceptual abnormalities. Several candidate genes for involvement in this disorder have emerged from patient studies, including *SHANK3*, a gene that encodes a protein (SHANK3) in the postsynaptic density of excitatory synapses. Previous work has shown that mutant mice carrying a human ASD mutation in the *Shank3* gene (*InsG3680*) exhibit repetitive behaviors and social interaction deficits, indicating important construct and face validity for this genotype as an animal model of ASD.

**Methods:**

To further address whether these mice also present auditory sensory–perceptual alterations, we developed a novel behavioral test in which mice can choose between different soundscapes.

**Results:**

Our results reveal that, in comparison to wild-type mice, *Shank3* mutants display a strong behavioral preference toward silent regions of the arena.

**Discussion:**

These data suggest that *Shank3*- mutant mice might express an auditory hypersensitivity phenotype, further adding to the face validity of this genotype as an animal model of ASD.

## 1. Introduction

Autism spectrum disorder (ASD) is a neurodevelopmental condition with a wide range of symptoms and degrees of severity that causes significant functional impairment in daily life (Mei et al., 2016; Robertson and Baron-Cohen, 2017). Updated prevalence estimates indicate that approximately 1 in 100 children are affected worldwide, with the prevalence of ASD being higher in boys (Zeidan et al., 2022). ASD is mainly characterized by difficulties in social communication and interaction across multiple contexts, and by repetitive behaviors, interests, or activities (American Psychiatric Association, 2013). Among the features of the restricted/repetitive behavior usually present in ASD are sensory alterations, such as unusual interest in sensory aspects of the environment or adverse response to specific sounds or textures (American Psychiatric Association, 2013; Robertson and Baron-Cohen, 2017). Studies have shown that hyperacusis (increased sensitivity or decreased tolerance to sound) can be found in 18–40% of children with ASD (American Psychiatric Association, 2013; Sinclair et al., 2017; WHO, 2018), possibly causing a wide range of cognitive and behavioral impairments (Gonçalves and Monteiro, 2023). Despite this evidence, the field still has a poor understanding of the neurobiological mechanisms that underlie auditory sensory–perceptual alterations in ASD and their contribution to social impairments.

In recent years, several studies have shown that behavioral alterations described in ASD patients can be closely recapitulated in animal models (Pasciuto et al., 2015; Schroeder et al., 2017; Zhou Y. et al., 2019; Siemann et al., 2020; Castro and Monteiro, 2022; Ivashko-Pachima et al., 2022). One of the most widely studied animal models of ASD is the *Shank3*-mutant mouse (Monteiro and Feng, 2017). *SHANK3* is a putative ASD-associated gene that encodes a scaffolding protein (SHANK3) enriched in the postsynaptic density of excitatory synapses (Monteiro and Feng, 2017; Monteiro, 2018). Previous studies by us and others have shown that rodent models carrying *Shank3* mutations exhibit various degrees of synaptic dysfunction and autistic-like behaviors, such as impaired social interaction, anxiety, and repetitive self-grooming (Bozdagi et al., 2010; Peça et al., 2011; Wang et al., 2011; Mei et al., 2016; Zhou et al., 2016; Monteiro and Feng, 2017; Monteiro, 2018; Song et al., 2019; Möhrle et al., 2020; Delling and Boeckers, 2021). Recently, with the aim of improving translational construct validity, a new mouse model was generated, specifically carrying a mutation found in a human ASD patient: the Shank3*G3680 mouse model of ASD (herein, such mice are referred to as *Shank3* KO mice) (Zhou et al., 2016). These transgenic mice lack SHANK3 expression due to a frameshift and downstream stop codon; they display ASD-like behaviors such as stereotypies, reduced social interaction, and impaired motor coordination, representing important construct and face validity for the model. Although well-established behavioral phenotyping assays exist for social deficits and repetitive patterns of behavior or interests in mice, the development of sensitive behavioral testing paradigms to assess sensorial deficits remains a challenging task (Wöhr, 2014). To further address whether these mice present with auditory sensory–perceptual alterations, we developed a novel test to assess auditory preference/avoidance at the behavioral level. Our results demonstrate that *Shank3* KO mice display a strong behavioral preference for silent environments, suggesting heightened auditory sensitivity, which corroborates findings from human ASD studies.

## 2. Materials and methods

### 2.1. Animals

All experimental procedures were approved by the local authorities of the Direção-Geral de Alimentação e Veterinária (ID: DGAV 8519) and the Ethics Subcommittee for the Life Sciences and Health (SECVS) of the University of Minho (ID: SECVS 01/18) and performed in accordance with European Community Council Directives (2010/63/EU) and Portuguese law DL N° 113/2013 for the care and use of laboratory animals. Animals were housed in a controlled environment (12 h light/dark cycles with lights on at 8 AM; constant temperature of 22°C and 55% humidity) with *ad libitum* access to water and food (4RF21, Mucedola). Shank3*G3680 mice of C57B6/S129 mixed genetic background were purchased from the Jackson Laboratory and kept in a Het × Het mixed background mating system. For use in the experiments, 92 Shank3*G3680 (KO) and 86 wild-type (WT) littermates were housed by sex and genotype with 2–5 mice per cage; all were aged 8–10 weeks at the time of the experiments.

### 2.2. Behavior

Behavioral tests were performed in darkness during the lights-off phase (8 PM) in a temperature-controlled room. Mice were permitted to acclimate to the behavioral test room for 1 h before the start of the test.

**FIGURE 1:**
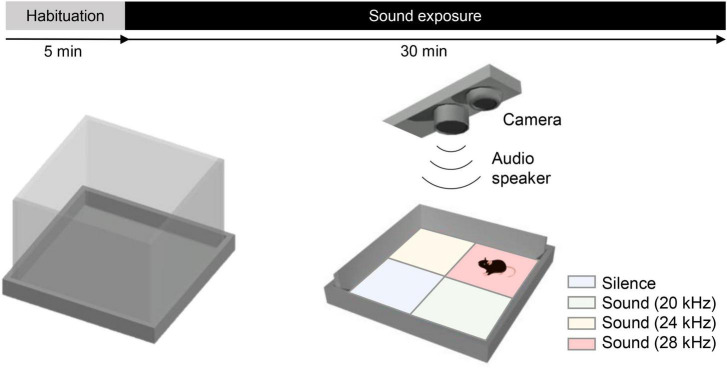
A novel behavioral test: the sound arena. Schematic shows the open field arena (44 × 44 × 30 cm) with an electrostatic speaker and a camera placed 60 cm above its center. The test duration was 35 min, consisting of 5 min of habituation and 30 min of exposure to tones. The arena was divided into four virtual quadrants (soundscapes): a silent quadrant (Q1) and three other quadrants associated with specific tones, such as Q2 = 20 kHz, Q3 = 24 kHz, and Q4 = 28 kHz. Tones associated with each virtual quadrant and the location of the silent quadrant were kept constant for each WT–KO pair and counterbalanced between pairs to minimize any potential bias.

The behavioral arena consisted of a custom-made open field (44 × 44 × 30 cm), with the floor covered with clean bedding (Figure 1). A free-field electrostatic speaker (ES1, TDT) (Table 1) driven by an electrostatic speaker driver (ED1, TDT) was placed 60 cm above the center of the arena to deliver specific tones to the arena. Mice were filmed at 20 fps using a camera (Flea3 USB3, Teledyne FLIR LLC) also placed 60 cm above the center of the arena, and the animals’ center of mass was tracked in real time using Bonsai (Lopes et al., 2015; Lopes and Monteiro, 2021). The arena was divided into four virtual quadrants (Q1–4), with three quadrants (Q2–4) associated with specific tones and one quadrant (Q1) associated with silence. The animal undergoing testing was allowed to freely explore the arena, and depending on its location (i.e., in which virtual quadrant the animal was located), a specific tone was played into the arena. Tones delivered (when the animal was in Q2–Q4) were either in the human-audible range (8, 12, and 16 kHz) or in the ultrasound range (20, 24, and 28 kHz). Sound intensity was measured at the bottom of the arena, and several intensities within a range of 50–80 dB SPL were tested, as also used by Manohar et al. (2017). No tones or other sound stimuli were delivered while the animal was located in the virtual quadrant associated with silence (Q1). To avoid place preference bias, the tones associated with each virtual quadrant and the location of the silent quadrant were kept constant for each WT–KO pair but randomized between different pairs of mice.

**TABLE 1:** Materials and methods.

Resource	Source	Identifier
**Mouse strain**		
Shank3*G3680 mice	Jackson Laboratory	028778
**Software**		
Matlab v2020b	MathWorks	https://www.mathworks.com/products/matlab.html
Bonsai v2.6.3	Bonsai-RX	https://bonsai-rx.org/
Prism 8	GraphPad	https://www.graphpad.com/
**Hardware**		
Flea3 USB 3 Camera	Teledyne FLIR LLC	FL3-U3-13Y3M-C
Free-field electrostatic speaker	Tucker-Davis Technologies	ES1
Sound card	Champalimaud Foundation	HARP
Electrostatic speaker driver	Tucker-Davis Technologies	ED1

Each test began with the animal being placed in the center of the arena, and experiment lasted for a total of 35 min per animal. The first 5 min were for arena exploration and habituation, and no tones were delivered regardless of the animal’s position. After the initial 5 min, the speaker began to deliver the tones continuously as described above, depending on the virtual quadrant in which the animal was located. Hence, as animals moved around the arena, different tones were played according to those associated with each quadrant (Figure 1). Pairs of naïve WT and KO littermates were tested on the same day.

### 2.3. Statistical analysis

The statistical analysis is detailed in Supplementary Table 1. Depending on the experimental design, either a two-tailed Student’s *t*-test or a one- or two-way ANOVA (with *post-hoc* tests for multiple comparisons) was used to assess the effect of genotype and other independent variables (e.g., sound frequency) on the dependent variable of interest. Differences between groups were considered statistically significant when *p* < 0.05. Data are expressed in the form of mean ± CI. Analyses were performed using Matlab v2020b (Mathworks) and GraphPad Prism (GraphPad Software Inc.).

## 3. Results

### 3.1. The sound arena behavioral test

One of the limiting factors in addressing potential auditory sensitivity issues in rodent models of ASD is the scarcity of auditory behavioral tests. The acoustic startle response (ASR) test measures the rapid contraction of facial and skeletal muscles in response to an abrupt, intense, unexpected auditory stimulus (i.e., a startling stimulus). The pre-pulse inhibition (PPI) test measures attenuation of the ASR by the presentation of a low-intensity pre-stimulus (pre-pulse) immediately preceding the startling stimulus. Some studies have used the PPI test or modified versions of ASR procedures as behavioral readouts of auditory function or auditory-guided behavior in rodents (Lauer et al., 2017). Previous results have shown that Shank3*G3680 mice display significantly reduced PPI (decreased attenuation), which may suggest the presence of auditory–perceptual alterations (Zhou et al., 2016). However, behavioral outcomes from the PPI test rely not only on auditory processing but also on the sensorimotor gating response. To better address auditory-dependent behavior in *Shank3* KO mice, we developed a novel paradigm that tests auditory preference/avoidance at a behavioral level: the sound arena. This paradigm consists of an open-field arena divided into four virtual quadrants or soundscapes: one silent, and three others associated with specific tones. Depending on the animal’s location, tracked in real time, a specific tone is played, or no sound is played (if the animal is located in the silent quadrant). The animal is first placed in the center of the arena and allowed to freely explore for 5 min (with no exposure to sound). After these initial 5 min, the speaker begins to deliver a specific tone depending on the quadrant where the animal is located. In the present study, tones delivered were either in the human-audible range (8, 12, and 16 kHz) or in the ultrasound range (20, 24, and 28 kHz). Each experiment lasted 35 min per animal: 5 min for arena exploration and habituation, plus 30 min of testing. For data analysis purposes, the quadrant associated with silence is always labeled Q1, the 20- or 8-kHz quadrant is Q2, the 24- or 12-kHz quadrant is Q3, and the 28- or 16-kHz quadrant is Q4.

**FIGURE 2:**
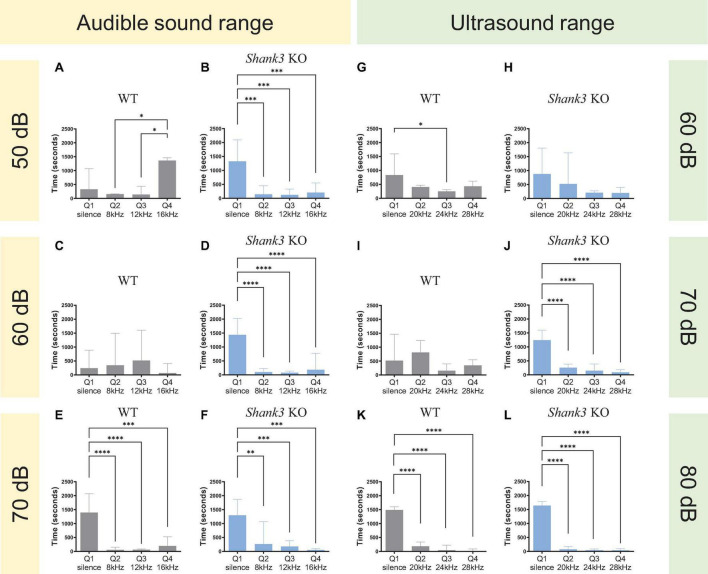
Sound intensity thresholds for *Shank3* KO mice in the audible and ultrasound range. **(A,B)** *Shank3* KO, but not WT mice, avoided quadrants associated with the delivery of auditory tones at 8, 12, and 16 kHz at 50 dB SPL [*n* = 3 WT mice, *F*(3,8) = 6.552, *p* = 0.0151; *n* = 3 *Shank3* KO mice, *F*(3,8) = 30.14, *p* = 0.0001]. **(C,D)** *Shank3* KO, but not WT mice, avoided quadrants associated with the delivery of auditory tones at 8, 12, and 16 kHz at 60 dB SPL [*n* = 3 WT mice, *F*(3,8) = 0.7766, *p* = 0.5392; *n* = 3 *Shank3* KO mice, *F*(3,8) = 45.70, *p* < 0.0001]. **(E,F)** Both *Shank3* KO and WT mice avoided quadrants associated with the delivery of auditory tones at 8, 12, and 16 kHz at 70 dB SPL [*n* = 3 WT mice, *F*(3,8) = 36.43, *p* < 0.0001; *n* = 3 *Shank3* KO mice, *F*(3,8) = 23.85, *p* = 0.0002]. **(G,H)** *Shank3* KO and WT mice did not display a strong quadrant preference in an arena delivering ultrasound tones at 20, 24, and 28 kHz at 60 dB SPL [*n* = 4 WT mice, *F*(3,12) = 3.636, *p* = 0.0449; *n* = 3 *Shank3* KO mice, *F*(3,8) = 3.591, *p* = 0.0658]. **(I,J)** *Shank3* KO, but not WT mice, avoided quadrants associated with the delivery of auditory tones at 20, 24, and 28 kHz at 70 dB SPL [*n* = 4 WT mice, *F*(3,12) = 1.323, *p* = 0.3126; *n* = 4 *Shank3* KO mice, *F*(3,12) = 57.76, *p* < 0.0001]. **(K,L)** Both *Shank3* KO and WT mice avoided quadrants associated with the delivery of auditory tones at 20, 24, and 28 kHz at 80 dB SPL [*n* = 4 WT mice, *F*(3,12) = 243.3, *p* < 0.0001; *n* = 6 *Shank3* KO mice, *F*(3,20) = 470.8, *p* < 0.0001]. All graphs plot the mean ± CI. Asterisks indicate a significant difference in a one-way ANOVA with Tukey’s multiple comparisons test: *p < 0.05, **p < 0.01, ***p < 0.001, ****p < 0.0001.

As the mice used in this study were a mouse model of ASD, it was crucial to identify appropriate sound intensity levels. Hence, prior to conducting the experiment, an optimization phase was carried out to determine the minimum sound intensities at which differences could be observed in the KO mice while ensuring no discernible effects on the behavior of WT mice (Figure 2).

For sounds in the audible range (8, 12, and 16 kHz), the results revealed that a sound intensity of 50 dB SPL (sound pressure level) was sufficient to observe differences in the behavior of KO mice but not WT mice [Figure 2A, *n* = 3 WT mice, *F*(3,8) = 6.552, *p* = 0.0151; Figure 2B, *n* = 3 *Shank3* KO mice, *F*(3,8) = 30.14, *p* = 0.0001]. In contrast, for sounds in the ultrasound range (20, 24, and 28 kHz), a sound intensity of 70 SPL was the minimum threshold at which significant differences could be observed at the behavioral level between KO and WT mice [Figure 2I, *n* = 4 WT mice, *F*(3,12) = 1.323, *p* = 0.3126; Figure 2J, *n* = 4 *Shank3* KO mice, *F*(3,12) = 57.76, *p* < 0.0001].

Based on these results, we selected a sound intensity of 50 dB SPL for the audible version of the sound arena and 70 dB SPL for the ultrasound version of the sound arena as the best intensities to distinguish between genotypes.

### 3.2. *Shank3* KO but not WT mice displayed auditory avoidance in the sound arena behavioral test using stimuli in the ultrasound range at 70 dB SPL

**FIGURE 3:**
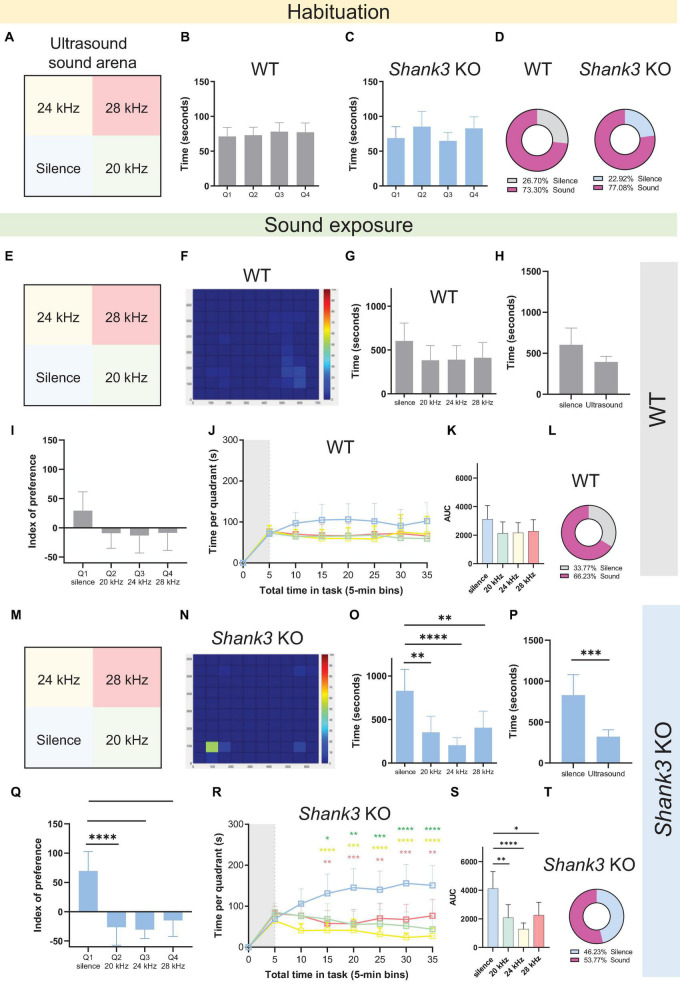
Male *Shank3* KO mice exhibit auditory avoidance in the sound arena behavioral test. **(A,E,M)** Representation of the four virtual quadrants (the soundscape) used in the experiment with ultrasound tones: a silent quadrant (Q1) and three other quadrants associated with specific tones, namely 20 kHz (Q2), 24 kHz (Q3), and 28 kHz (Q4), delivered at 70 dB SPL. **(B,C)** Summary bar graphs reveal no differences in the amount of time spent in each quadrant during the habituation phase in either WT or *Shank3* KO mice [*n* = 23 WT mice, *F*(3,88) = 0.2860, *p* = 0.8354; *n* = 25 *Shank3* KO mice, *F*(3,96) = 1.489, *p* = 0.2224]. **(D)** Percentage of the total time spent in the silent quadrant (gray for WT; blue for *Shank3* KO mice) versus all other quadrants (pink) during the habituation phase. **(F)** Representative heat map showing behavior of one WT mouse during the 30-min sound exposure phase of the sound arena behavioral test. **(G)** Summary bar graphs reveal no differences in the amount of time spent in each quadrant during the sound exposure phase by WT mice [*n* = 23 WT mice, *F*(3,88) = 1.492, *p* = 0.2222]. **(H)** Summary bar graphs show no differences between total time spent in the silent quadrant and average time spent in each of the sound quadrants for WT mice [*n* = 23 WT, *t*(44) = 2.010, *p* = 0.0506]. **(I)** Preference index analysis shows no quadrant preference in WT mice [*n* = 23 WT, *F*(3,88) = 1.947, *p* = 0.1279]. **(J)** Absence of quadrant preference over time for WT mice: first 5 min of habituation (gray shaded area) plus 30 min of sound exposure [*F*(3,88) = 1.378, *p* = 0.2547]. **(K)** Bar graphs show total area under the curve (AUC) for time spent in the silent quadrant compared to all other quadrants in WT mice [*n* = 23 WT, *F*(3,88) = 1.402, *p* = 0.2475]. **(L)** Percentage of the total time spent in the silent quadrant (gray) versus all other quadrants (pink) during the sound exposure phase (*n* = 23 WT mice). **(N)** Representative heat map showing behavior of one *Shank3* KO mouse during the 30-min sound exposure phase of the sound arena behavioral test. **(O)** Summary bar graphs reveal a preference for the silent quadrant compared to the quadrants associated with 20, 24, and 28 kHz tones in *Shank3* KO mice [*n* = 25 *Shank3* KO mice, *F*(3,96) = 8.761, *p* < 0.0001]. **(P)** Summary bar graphs show that *Shank3* KO mice spent more time in the silent quadrant compared with the average time spent in each of the sound quadrants [*n* = 25 *Shank3* KO mice, *t*(48) = 4.004, *p* = 0.0002]. **(Q)** Preference index analysis shows a preference for the silent quadrant in *Shank3* KO mice [*n* = 25 *Shank3* KO mice, *F*(3,96) = 12.55, *p* < 0.0001]. **(R)** Emergence of quadrant preference over time for *Shank3* KO mice: first 5 min of habituation (gray shaded area) plus 30 min of sound exposure. *Shank3* KO mice preferred silence over sounds [*n* = 25 *Shank3* KO mice, *F*(3,96) = 7.921, *p* < 0.0001]. This preference for silence over all other quadrants emerged 15 min after onset of the sound exposure phase. **(S)** Bar graphs show total AUC for time spent in the silent quadrant compared to all other quadrants by *Shank3* KO mice [*n* = 25 *Shank3* KO mice, *F*(3,96) = 7.567, *p* = 0.0001]. **(T)** Percentage of the total time spent in the silent quadrant (blue) versus all other quadrants (pink) during the sound exposure phase (*n* = 25 *Shank3* KO mice). All graphs plot the mean ± CI. Asterisks indicate significant differences in a one-way ANOVA with Tukey’s multiple comparisons tests (panels **B,C,G,I,K,O,Q,S**), a two-way RM ANOVA with Tukey’s multiple comparisons tests (panels **J,R**), or a two-tailed unpaired *t*-test (panels **H,P**), *p < 0.05, **p < 0.01, ***p < 0.001, ****p < 0.0001.

Given that mice communicate via ultrasonic vocalizations (USVs), three frequencies were played in the ultrasound range: 20, 24, and 28 kHz (Figure 3A). The results revealed that, during the habituation phase, neither WT nor *Shank3* KO male mice exhibited any differences in the amount of time spent in each quadrant [Figure 3B, *F*(3,88) = 0.2860, *p* = 0.8354; Figure 3C, *F*(3,96) = 1.489, *p* = 0.2224], spending approximately 25% of the total time in each quadrant (Figure 3D). However, during the testing phase (Figures 3E–T), *Shank3* KO mice exhibited a significant preference for silence [Figure 3O, *F*(3,96) = 8.761, *p* < 0.0001], spending more time in the Q1 (silent) quadrant compared to all other quadrants [Figure 3P, *t*(48) = 4.004, *p* = 0.0002]. This preference was observed regardless of whether the quadrants were virtual or delimited by physical boundaries (Supplementary Figure 1). In an analysis of the individual index of preference (the individual time spent in each quadrant during sound exposure, subtracting the time spent in that quadrant during habituation phase), *Shank3* KO mice once again displayed a clear preference for the silent quadrant [Figure 3Q, *F*(3,96) = 12.55, *p* < 0.0001], spending approximately 46% of their time in that quadrant (Figure 3T) versus 33% of the total time spent in that quadrant by WTs (Figure 3L). The emergence of quadrant preference over time was also evaluated, with time divided into 5-min bins; the results showed that the preference for silence observed in *Shank3* KO mice emerged 15 min after the onset of sound exposure (Figure 3R). These results indicate that when mice can choose between different soundscapes, *Shank3* KO mice prefer silent environments to environments associated with tones in the ultrasound frequency range delivered at 70 dB SPL.

### 3.3. Locomotor activity in the sound arena

Given that reduced locomotor activity has been previously reported in *Shank3* KO mice, the total distance traveled in the sound arena was also evaluated (Supplementary Figures 2A–N). No differences between WT and *Shank3* KO mice were found in terms of locomotion (total distance traveled) during the habituation time (Supplementary Figures 2A–G), alongside a lack of preference for any of the locations within the arena (Supplementary Figures 2B, E). Analysis of the sound exposure period revealed no differences between the arena locations in terms of total distance traveled across them by WT mice [Supplementary Figure 2I, *F*(3,88) = 0.8238, *p* = 0.4842]. In contrast, *Shank3* KO mice exhibited more locomotion within the silent quadrant [Supplementary Figure 2L, *F*(3,96) = 6.198, *p* = 0.0007], which was also the quadrant where they spent most of their time.

### 3.4. Neither WT nor *Shank3* KO mice displayed auditory avoidance in the sound arena behavioral test using stimuli in the human-audible range at 50 dB SPL

**FIGURE 4:**
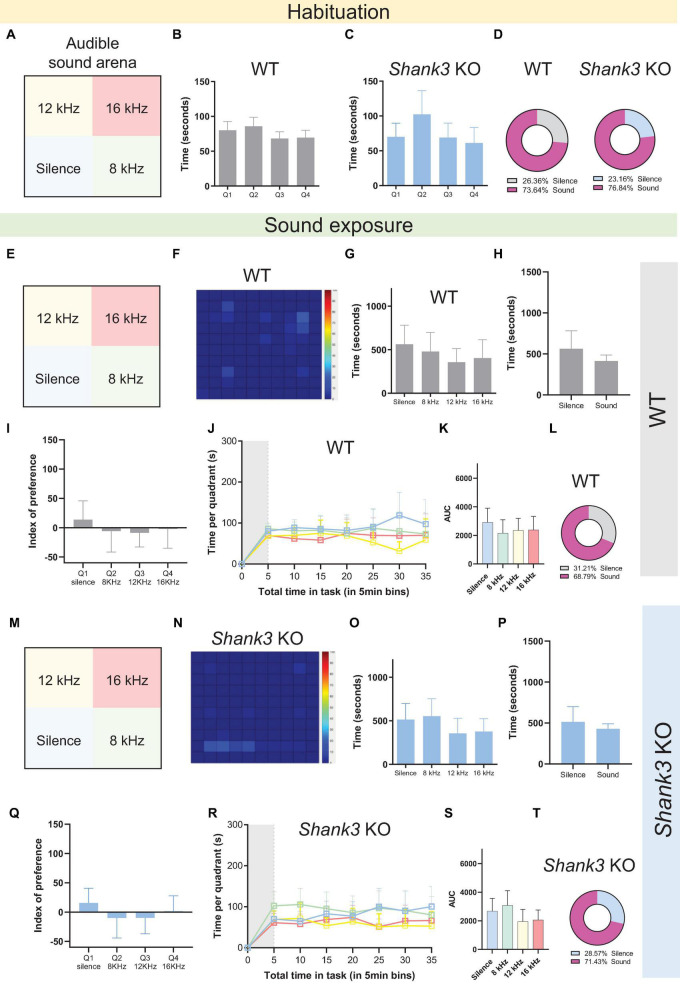
Wild-type and *Shank3* KO mice do not show auditory avoidance to tones in the human audible range at 50 dB SPL. **(A,E,M)** Representation of the four virtual quadrants (the soundscape) used in the experiment with audible sounds: a silent quadrant (Q1) and three other quadrants associated with specific tones, namely 8 kHz (Q2), 12 kHz (Q3), and 16 kHz (Q4), delivered at 50 dB SPL. **(B,C)** Summary bar graphs reveal no differences in the amount of time spent in each quadrant during the habituation phase in either WT or *Shank3* KO mice [*n* = 19 WT mice, *F*(3,72) = 2.387, *p* = 0.0760; *n* = 20 *Shank3* KO mice, *F*(3,76) = 2.354, *p* = 0.0786]. **(D)** Percentage of the total time spent in the silent quadrant (gray for WT; blue for *Shank3* KO mice) versus all other quadrants (pink) during the habituation phase. **(F)** Representative heat map showing behavior of one WT mouse during the 30-min sound exposure phase of the sound arena behavioral test. **(G)** Summary bar graphs reveal no differences in the amount of time spent in each quadrant during the sound exposure phase by WT mice [*n* = 19 WT mice, *F*(3,72) = 0.8715, *p* = 0.4600]. **(H)** Summary bar graphs show no differences between total time spent in the silent quadrant and average time spent in each of the sound quadrants for WT mice [*n* = 19 WT, *t*(36) = 1.362, *p* = 0.1817]. **(I)** Preference index analysis shows no quadrant preference in WT mice [*n* = 19 WT, *F*(3,72) = 0.4515, *p* = 0.7170]. **(J)** Absence of quadrant preference over time for WT mice: first 5 min of habituation (gray shaded area) plus 30 min of sound exposure [*n* = 19 WT, *F*(3,72) = 0.9625, *p* = 0.4152]. **(K)** Bar graphs show total area under the curve (AUC) for time spent in the silent quadrant compared to all other quadrants in WT mice [*n* = 19 WT, *F*(3,72) = 0.5335, *p* = 0.6608]. **(L)** Percentage of the total time spent in the silent quadrant (gray) versus all other quadrants (pink) during the sound exposure phase (*n* = 19 WT mice). **(N)** Representative heat map showing behavior of one *Shank3* KO mouse during the 30-min sound exposure phase of the sound arena behavioral test. **(O)** Summary bar graphs reveal no differences in the amount of time spent in each quadrant during the sound exposure phase in *Shank3* KO mice [*n* = 20 *Shank3* KO mice, *F*(3,76) = 1.365, *p* = 0.2599]. **(P)** Summary bar graphs show no differences between total time spent in the silent quadrant and average time spent in each of the sound quadrants for *Shank3* KO mice [*n* = 20 *Shank3* KO mice, *t*(38) = 0.9237, *p* = 0.3615]. **(Q)** Preference index analysis shows no quadrant preference in *Shank3* KO mice [*n* = 20 *Shank3* KO mice, *F*(3,76) = 0.8080, *p* = 0.4933]. **(R)** Absence of quadrant preference over time for *Shank3* KO mice: first 5 min of habituation (gray shaded area) plus 30 min of sound exposure [*n* = 20 *Shank3* KO mice, *F*(3,76) = 1.582, *p* = 0.2008]. **(S)** Bar graphs show total (AUC) for time spent in the silent quadrant compared to all other quadrants in *Shank3* KO mice [*n* = 20 *Shank3* KO mice, *F*(3,76) = 1.629, *p* = 0.1897]. **(T)** Percentage of the total time spent in the silent quadrant (blue) versus all other quadrants (pink) during the sound exposure phase (*n* = 20 *Shank3* KO mice). All graphs plot the mean ± CI. Asterisks indicate significant differences in a one-way ANOVA with Tukey’s multiple comparisons tests (panels **B,C,G,I,K,O,Q,S**), a two-way RM ANOVA with Tukey’s multiple comparisons tests (panels **J,R**), or a two-tailed unpaired *t*-test (panels **H,P**).

Although mice communicate with one another via USVs, their non-optimal hearing range extends into the human-audible range, down to approximately 1 kHz (Heffner and Heffner, 2007). Thus, we decided to further test whether the auditory avoidance phenotype observed in *Shank3* KO mice could also be observed for frequencies in the human-audible range. Accordingly, we conducted a new version of the sound arena behavioral test using three different tones within the human audible range: 8, 12, and 16 kHz, delivered at 50 dB SPL (Figures 4A–T). A new cohort of naïve male *Shank3* KO and WT littermates was used for behavioral testing. The results revealed no significant differences for either WT or *Shank3* KO mice, either during the habituation phase (Figures 4B, C and Supplementary Table 1) or during the testing phase (Figures 4E–T and Supplementary Table 1). These results indicate that the behavioral avoidance displayed by *Shank3* KO mice for ultrasound stimuli, as illustrated in Figure 3, can be attributed either to differences in the intensity of the sounds used in each test (50 dB vs. 70 dB SPL) or to specific auditory avoidance of sounds in the ultrasound range, in which the peak hearing range of the mice is found and which is closer to their preferred range for communication via vocalizations (Zippelius and Schleidt, 1956; Premoli et al., 2019).

## 4. Discussion

Several studies using electrophysiology and brain imaging data from ASD patients have revealed significant changes in sensory stimuli processing (Mamashli et al., 2017; Stickel et al., 2019; Pierce et al., 2021), specifically involving altered auditory perceptual capacity and deficits in auditory discrimination tasks, especially in the presence of noise (Gonçalves and Monteiro, 2023). In accordance with these findings, auditory alterations have been reported across several animal models of ASD, such as hyperacusis in *Fmr1*-KO rodents (Auerbach et al., 2021; Wilde et al., 2022), increased pitch discrimination abilities in *Shank3B*-KO mice (Rendall et al., 2019), increased thresholds for tone-evoked cortical responses in *Mecp2* transgenic mice (Zhou C. et al., 2019), and auditory processing impairments in *Cntnap2*-KO rodents (Scott et al., 2018) as well as several *Shank3* transgenic models (Castro and Monteiro, 2022). Our results expand these observations by revealing an auditory behavioral avoidance phenotype in *Shank3* KO mice carrying a human ASD mutation in the *Shank3* gene.

Mice communicate with each other in the ultrasonic range, shaping social dynamics and conveying emotional states at frequencies above the range of human hearing (>20 kHz) (Zippelius and Schleidt, 1956; Premoli et al., 2019). Although their hearing ranges from approximately 1 to 100 kHz (Heffner and Heffner, 2007), they have poor low-frequency hearing and display peak sensitivity at around 20 kHz (Reynolds et al., 2010; Ehret and Riecke, 2022). Accordingly, we developed a new behavioral test (the sound arena) that allows mice to freely move between different soundscapes associated with tones ranging from 8 to 16 kHz at 50 dB SPL and from 20 to 28 kHz at 70 dB SPL. The results revealed that *Shank3* KO mice avoid soundscapes associated with all the tested ultrasound frequencies (20, 24, and 28 kHz) at 70 dB and seek shelter in silent areas of the arena, suggesting an auditory avoidance phenotype in comparison to WT mice. However, it is important to mention that caution should be exercised in the interpretation of potential differences between genotypes. Although a clear trend appears to be present in KO vs. WT mice regarding the total time spent in the silent quadrant, these differences between groups were not significant. Significant differences were only observed within the *Shank3* KO group in relation to the amount of time these mice spent in the silent quadrant vs. total time in all other quadrants. These findings indicate that under the experimental conditions described (access to one silent quadrant and three other quadrants associated with ultrasound frequencies delivered at 70 dB), *Shank3* KO mice demonstrate auditory avoidance behavior and actively withdraw to the silent quadrant. Comparatively, under the same experimental conditions, WT mice do not exhibit significant preference for any quadrant. This suggests that *Shank3* KO mice might have altered auditory sensory perception and highlights the relevance of further investigating the specific mechanisms underlying this behavior in the context of the *Shank3-InsG3680(*+/+) mouse model of ASD.

Impairments of auditory sensory processing may impact auditory filtering and the ability to focus on relevant sensory cues (awareness/distractibility), impacting social interaction in mice and mirroring social communication deficits present in ASD patients. Uncovering auditory-driven behavioral phenotypes in mice can be challenging, and most studies have focused on functional (rather than behavioral) auditory phenotypes.

One such example of functional testing is the auditory brainstem response (ABR) test, typically used to assess hearing thresholds and the integrity of the auditory neural pathway. However, no consistent differences in ABR thresholds have been found between rodent models of ASD and WT animals (Wilde et al., 2022). Regarding auditory behavioral tests, these typically tend to be related to conditioned/unconditioned or reflexive responses to sound. Furthermore, behavioral tests requiring animal conditioning, such as GO/NO GO tasks or conditioned suppression/avoidance (Willott, 2001), involve other brain processes such as attention and valence, making them often not specific tests of purely auditory-driven behavior. Unconditioned or reflexive response tests such as the ASR and the PPI test have previously been performed in *Shank3*-mutant mice, revealing an increased ASR and decreased PPI (Zhou et al., 2016; Monteiro and Feng, 2017). Such results hint at a possible auditory hypersensitivity phenotype in *Shank3*-mutant mice, a finding that is well aligned with the behavioral results that we have observed in our sound arena test.

Of note, a number of previous studies have investigated sound processing abilities using rat models. These studies have focused on assessing the perceptual ability to categorize the intensity of sounds (subjectively, i.e., their loudness), as well as assessing sound avoidance behavior [via the active sound avoidance paradigm (ASAP), in which rats can choose a place context in response to variations in sound intensity] (Manohar et al., 2017; Scott et al., 2020). Scott et al. (2020) tested the ability of an ASD rat model that was trained to discriminate between loud (89 dB SPL) and quiet (71 dB SPL) stimuli to categorize the intensity of sounds. Notably, no differences between genotypes were found in terms of objective sound categorization abilities, suggesting typical sound intensity categorization in the ASD rat model (intact hearing). Interestingly, the same authors used an ASAP to assess putative aversion to moderate-intensity sounds and found that animals in the ASD group exhibited avoidance behavior at lower sound levels, a result that mirrors our own observations well. These converging results indicate a potential shared auditory hypersensitivity phenotype in both the rat ASD model and the *Shank3*-mutant mouse model of ASD. Both paradigms (the ASAP and our novel sound arena behavioral test) can provide valuable information, as they enable the measurement of preferences for different sound intensities without requiring previous training, memory, or learning skills in the subjects. Nevertheless, given that the test requires mice to associate specific locations within the arena with different acoustic stimuli, it is important to consider the potential relationship between the experimental framework and their learning abilities. Although our study did not directly assess the learning abilities of *Shank3*-mutant mice, previous research has shown that different *Shank3* mutations can impact learning and memory (Monteiro and Feng, 2017). Therefore, future research could focus on comprehensive assessments of learning and memory, including spatial and associative learning paradigms, to fully understand the impact of the *Shank3-InsG3680(*+/+) mutation on learning abilities, particularly in the context of auditory processing.

### 4.1. Experimental limitations and opportunities for future work

The auditory alterations described in ASD may be caused by bottom-up or top-down deficits in sensory processing, likely involving changes in brain structures involved in higher-level processing, such as the auditory cortex. Regarding the observed auditory-driven behavioral changes described in our work and considering other studies showing normal ABR in rodent models of ASD, it is likely that the behavioral changes observed in our study are due to alterations in central auditory processing rather than peripheral processing. As such, future studies should disentangle these two possibilities and explore the putative neuronal mechanisms underlying these behavioral symptoms.

Given the higher prevalence of ASD in boys, at a ratio of 4:1 (Zeidan et al., 2022), and to avoid the potentially confounding variable of animal sex in our study, we carried out our experiments in male *Shank3* KO mice at approximately 8–10 weeks of age. Accordingly, our conclusions are limited to this experimental group. To thoroughly investigate the auditory avoidance phenotype, it is important for future studies to include female mice as well.

Despite these and other possible limitations, our results demonstrate that the *Shank3-InsG3680(+/+)* mouse model of ASD displays auditory avoidance and points to the need for future research on auditory circuit dysfunctions associated with disorders falling within the autism spectrum.

## Data availability statement

The original contributions presented in this study are included in the article/Supplementary material, further inquiries can be directed to the corresponding author.

## Ethics statement

The animal study was approved by the Direção-Geral de Alimentação e Veterinária (ID: DGAV 8519) and the Ethics Subcommittee for the Life Sciences and Health (SECVS) of the University of Minho (ID: SECVS 01/18). The study was conducted in accordance with the local legislation and institutional requirements.

## Author contributions

AG performed all experiments, conceptualized the study, and wrote the manuscript. NS, LJ, and PM conceptualized the study and revised the manuscript. All authors contributed to the article and approved the submitted version.
