# Supplementary material for: The Shank3-InsG3680(+/+) mouse model of autism spectrum disorder displays auditory avoidance in a novel behavioral test
Source: Front Behav Neurosci. 2023 Aug 24;17:1205507. doi: 10.3389/fnbeh.2023.1205507 (PMC10483143; doi:10.3389/fnbeh.2023.1205507)
Supplement: Supplementary file 1 [file Data_Sheet_1.PDF]

Supplementary Material

Ana Margarida Gonçalves, Nuno Sousa, Luis Jacinto, Patricia Monteiro*

*** Correspondence:** Corresponding Author: pmonteiro@med.up.pt

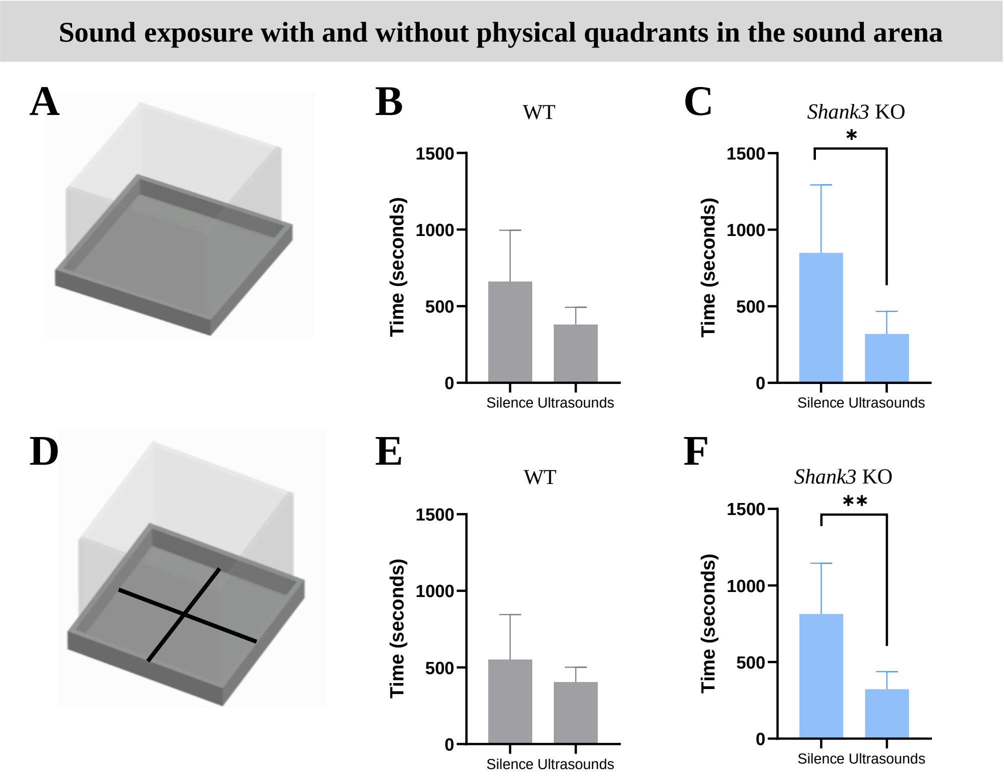

Supplementary Figure 1 | Experiment with virtual vs physical quadrants. A. Illustration of the sound arena without physical barriers between the quadrants. **B.** Summary bar graphs show no differences between the quadrant of silence and the average time spent in the sound quadrants for WT mice (n = 11 WT, t (20) =1.758, P=0.0940). **C.** Summary bar graphs show a preference for the silence quadrant compared with the average time spent in the sound quadrants for *Shank3* KO mice (n = 11 *Shank3* KO, t (20) = 2.526, P=0.0201). **D.** Illustration of the sound arena with a cross at the base of the arena, creating physical quadrants. **E.** Summary bar graphs show no differences between the quadrant of silence and the average time spent in the sound quadrants for WT mice (n = 12 WT, t (22) = 1.027, P= 0.3157). **F.** Summary bar graphs show a preference for the silence quadrant compared with the average time spent in the sound quadrants for *Shank3* KO mice (n = 14 *Shank3* KO, t (26) =3.020, P=0.0056). Data are mean ± CI. Unpaired t-tests; *$p < 0.05$, **$p < 0.01$.

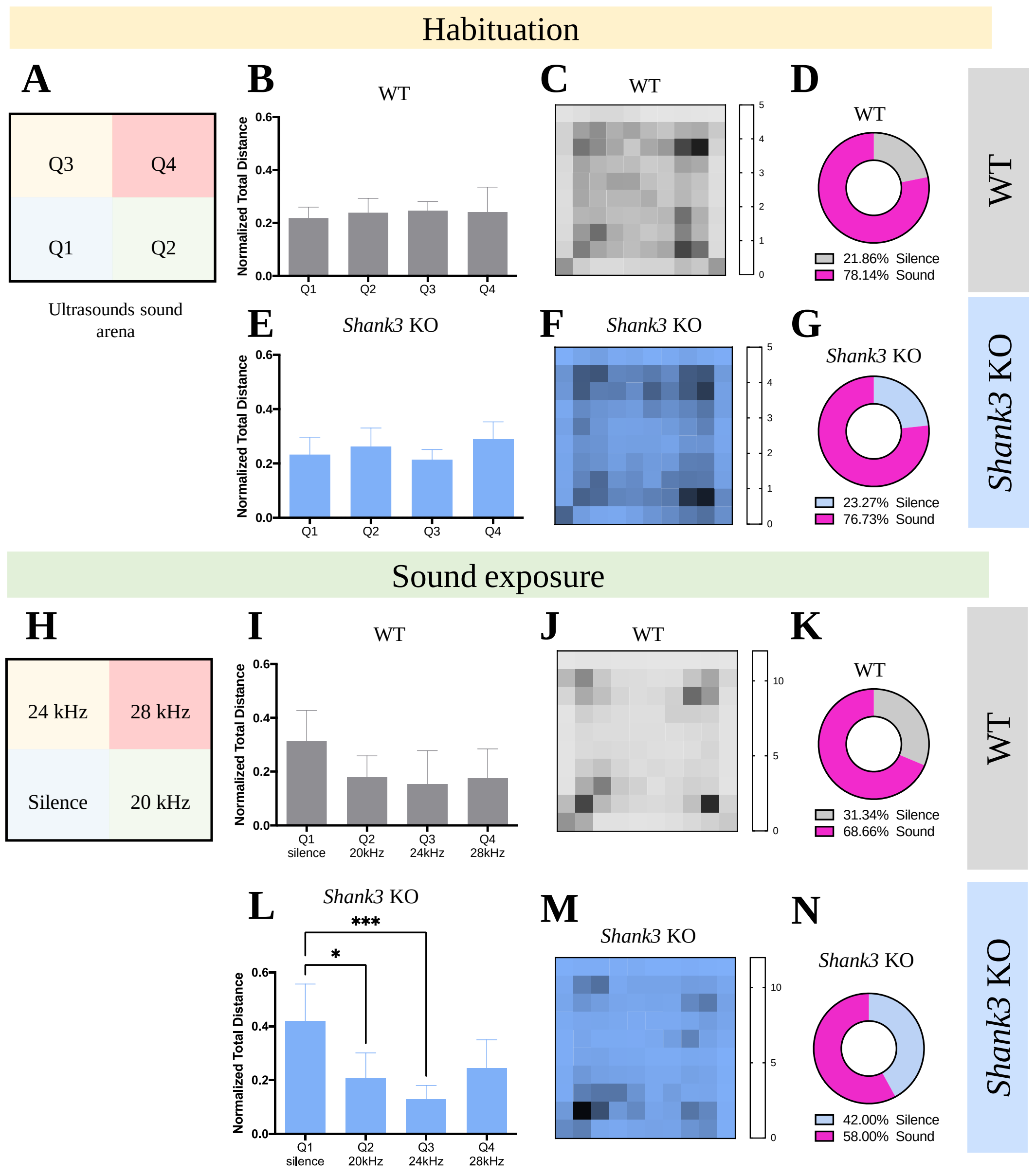

Supplementary Figure 2 | Sound arena locomotor activity. A. Representation of four virtual quadrants (soundscapes) of the experiment in the habituation period. **B.** Summary bar graphs reveal no differences in normalized total distance traveled in the arena during the 5 min of habituation (*n* = 23 WT, F (3, 88) = 1.465, P=0.2297). **C.** Heatmap of WT mouse in the sound arena during the 5 min of habituation. **D.** Percentage of traveled distance at the quadrant of silence (grey) and the average of traveled distance at the quadrants of sound (pink) during the habituation period (*n* = 23 WT). **E.** Summary bar graphs reveal no differences in normalized total distance traveled in the arena during the 5 min of habituation (*n* = 25 *Shank3* KO mice, F (3, 96) = 1.394, P=0.2495). **F.** Heatmap of *Shank3* KO mouse in the sound arena during the 5 min of habituation. **G.** Percentage of traveled distance at the

quadrant of silence (blue) and the average of traveled distance at the quadrants of sound (pink) during the habituation period (*n* = 25 *Shank3* KO mice). **H.** Representation of four virtual quadrants (soundscapes) of the experiment with ultrasounds range: a silent quadrant (Q1) and three other quadrants associated with specific tones such as 20 kHz (Q2), 24 kHz (Q3), and 28 kHz (Q4), at 70dB SPL. **I.** Summary bar graphs reveal no differences in normalized total distance traveled in the arena during the sound exposure (*n* = 23 WT, $F(3, 88) = 0.8238$, $P=0.4842$). **J.** Heatmap of WT mouse in the sound arena during the sound exposure. **K.** Percentage of traveled distance at the quadrant of silence (grey) and the average of traveled distance at the quadrants of sound (pink) during the sound exposure period (*n* = 23 WT). **L.** Summary bar graphs reveal differences in the normalized total distance traveled in the arena during the sound exposure period (*n* = 25 *Shank3* KO mice, $F(3, 96) = 6.198$, $P=0.0007$). **M.** Heatmap of *Shank3* KO mouse in the sound arena during the sound exposure. **N.** Percentage of traveled distance at the quadrant of silence (blue) and the average of traveled distance at the quadrants of sound (pink) during sound exposure period (*n* = 25 *Shank3* KO mice).

Suppl.Table 1 - Statistical analysis

Figure	N	Test	Result
2A	WT=3	One-way ANOVA, Tukey's multiple comparisons test	F (3, 8) = 6.552, P=0.0151
2B	KO=3	One-way ANOVA, Tukey's multiple comparisons test	F (3, 8) = 30.14, P=0.0001
2C	WT=3	One-way ANOVA, Tukey's multiple comparisons test	F (3, 8) = 0.7766, P=0.5392
2D	KO=3	One-way ANOVA, Tukey's multiple comparisons test	F (3, 8) = 45.70, P<0.0001
2E	WT=3	One-way ANOVA, Tukey's multiple comparisons test	F (3, 8) = 36.43, P<0.0001
2F	KO=3	One-way ANOVA, Tukey's multiple comparisons test	F (3, 8) = 23.85, P=0.0002
2G	WT=4	One-way ANOVA, Tukey's multiple comparisons test	F (3, 12) = 3.636,P=0.0449
2H	KO=3	One-way ANOVA, Tukey's multiple comparisons test	F (3, 8) = 3.591, P=0.0658
2I	WT=4	One-way ANOVA, Tukey's multiple comparisons test	F (3, 12) = 1.323, P=0.3126
2J	KO=4	One-way ANOVA, Tukey's multiple comparisons test	F (3, 12) = 57.76, P<0.0001
2K	WT=4	One-way ANOVA, Tukey's multiple comparisons test	F (3, 12) = 243.3, P<0.0001
2L	KO=6	One-way ANOVA, Tukey's multiple comparisons test	F (3, 20) = 470.8, P<0.0001
3B	WT=23	One-way ANOVA, Tukey's multiple comparisons test	F (3, 88) = 0.2860, P=0.8354
3C	KO=25	One-way ANOVA, Tukey's multiple comparisons test	F (3, 96) = 1.489, P=0.2224
3G	WT=23	One-way ANOVA, Tukey's multiple comparisons test	F (3, 88) = 1.492, P=0.2222
3H	WT=23	Two-tailed unpaired t-test	*t* (44) = 2.010, P = 0.0506
3I	WT=23	One-way ANOVA, Tukey's multiple comparisons test	F (3, 88) = 1.947, P=0.1279
3J	WT=23	Two-way repeated measures ANOVA, Tukey's multiple comparisons test	Time*Quadrant: F (21, 616) = 0.9801, P=0.4863 ; Time: F (2.731, 240.3) = 27.67, P<0.0001; Quadrant: F (3, 88) = 1.378, P=0.2547
3K	WT=23	One-way ANOVA, Tukey's multiple comparisons test	F (3, 88) = 1.402, P=0.2475
3O	KO=25	One-way ANOVA, Tukey's multiple comparisons test	F (3, 96) = 8.761, P<0.0001
3P	KO=25	Two-tailed unpaired t-test	*t* (48) = 4.004, P=0.0002
3Q	KO=25	One-way ANOVA, Tukey's multiple comparisons test	F (3, 96) = 12.55, P<0.0001
3R	KO=25	Two-way repeated measures ANOVA, Tukey's multiple comparisons test	Time*Quadrant: F (21, 672) = 6.833, P<0.0001 ; Time: F (3.127, 300.2) = 32.16, P<0.0001; Quadrant: F (3, 96) = 7.921, P<0.0001
3S	KO=25	One-way ANOVA, Tukey's multiple comparisons test	F (3, 96) = 7.567, P=0.0001
4B	WT=19	One-way ANOVA, Tukey's multiple comparisons test	F (3, 72) = 2.387, P=0.0760
4C	KO=20	One-way ANOVA, Tukey's multiple comparisons test	F (3, 76) = 2.354, P=0.0786
4G	WT=19	One-way ANOVA, Tukey's multiple comparisons test	F (3, 72) = 0.8715, P=0.4600
4H	WT=19	Two-tailed unpaired t-test	*t* (36) = 1.362, P=0.1817
4I	WT=19	One-way ANOVA, Tukey's multiple comparisons test	F (3, 72) = 0.4515, P=0.7170
4J	WT=19	Two-way repeated measures ANOVA, Tukey's multiple comparisons test	Time*Quadrant: F (21, 504) = 1.117, P=0.3255; Time: F (2.526, 181.9) = 20.67, P<0.0001; Quadrant: F (3, 72) = 0.9625, P=0.4152
4K	WT=19	One-way ANOVA, Tukey's multiple comparisons test	F (3, 72) = 0.5335, P=0.6608
4O	KO=20	One-way ANOVA, Tukey's multiple comparisons test	F (3, 76) =1.365, P=0.2599
4P	KO=20	Two-tailed unpaired t-test	*t* (38) =0.9237,P= 0.3615
4Q	KO=20	One-way ANOVA, Tukey's multiple comparisons test	F (3, 76) = 0.8080, P=0.4933
4R	KO=20	Two-way repeated measures ANOVA, Tukey's multiple comparisons test	Time*Quadrant: F (21, 532) = 1.045, P=0.4061; Time: F (3.645, 277.0) = 19.86, P<0.0001; Quadrant: F (3, 76) = 1.582, P=0.2008
4S	KO=20	One-way ANOVA, Tukey's multiple comparisons test	F (3, 76) = 1.629, P=0.1897
Supplementary 1B	WT=11	Two-tailed unpaired t-test	*t* (20) =1.758, P=0.0940
Supplementary 1C	KO=11	Two-tailed unpaired t-test	*t* (20) =2.526, P=0.0201
Supplementary 1E	WT=12	Two-tailed unpaired t-test	*t* (22) = 1.027, P= 0.3157
Supplementary 1F	KO=14	Two-tailed unpaired t-test	*t* (26) =3.020, P=0.0056
Supplementary 2B	WT=23	One-way ANOVA, Tukey's multiple comparisons test	F (3, 88) = 1.465, P=0.2297
Supplementary 2E	KO=25	One-way ANOVA, Tukey's multiple comparisons test	F (3, 96) = 1.394, P=0.2495
Supplementary 2I	WT=23	One-way ANOVA, Tukey's multiple comparisons test	F (3, 88) = 0.8238, P=0.4842
Supplementary 2L	KO=25	One-way ANOVA, Tukey's multiple comparisons test	F (3, 96) = 6.198, P=0.0007
